# Nitric Oxide-Mediated Regulation of Chitinase Activity and Cadmium Sequestration in the Response of *Schizophyllum commune* to Cadmium Stress

**DOI:** 10.3390/microorganisms13030470

**Published:** 2025-02-20

**Authors:** Dongxu Li, Chen Chu, Mengshi Zhao, Suying Hou, Rong Ji, Changhong Liu

**Affiliations:** 1 State Key Laboratory of Pharmaceutical Biotechnology, School of Life Sciences, Nanjing University, Nanjing 210023, China; laolang_2012@163.com (D.L.); mg1930112@smail.nju.edu.cn (C.C.); 602022300055@smail.nju.edu.cn (M.Z.); 2 College of Life Sciences, Yunnan University, Kunming 650500, China; housuying1008@163.com; 3 State Key Laboratory of Pollution Control and Resource Reuse, School of Environment, Nanjing University, Nanjing 210023, China; ji@nju.edu.cn

**Keywords:** cadmium, nitric oxide, nitrate reductase, chitinase, *Schizophyllum commune*, heavy-metal stress

## Abstract

*Schizophyllum commune* is an edible fungus with high medicinal value, but exposure to heavy-metal pollution poses significant health risks. Cadmium (Cd) toxicity inhibits fungal growth and leads to Cd accumulation in the mycelium. However, the regulatory mechanisms of Cd-induced growth inhibition and Cd accumulation remain poorly understood. Here, *S. commune* 20R-7-F01 was cultured in Cd-supplemented minimal medium (MM) to investigate the response of *S. commune* 20R-7-F01 to Cd exposure. We found that Cd exposure resulted in growth inhibition and a Cd-dependent increase in endogenous nitric oxide (NO) levels. NO production was primarily mediated by the nitrate reductase (NR) pathway. Cd-induced growth inhibition was alleviated by inhibiting NR activity or scavenging NO, highlighting the role of NO in stress responses. Furthermore, NO was found to enhance chitinase activity, thereby promoting Cd accumulation in the fungal cell wall and leading to growth inhibition. These results reveal a novel mechanism by which *S. commune* copes with Cd stress. This study highlights the potential of manipulating NO levels as a strategy to enhance fungal tolerance to heavy-metal pollution, providing a new avenue for managing environmental stresses in edible fungi and protecting human health.

## 1. Introduction

Nitric oxide (NO) is a versatile signaling molecule that regulates a wide array of biological processes across plants, animals, and microorganisms [1,2,3]. In fungi, NO plays a pivotal role in mediating both physiological and stress-related responses [4,5] and has been implicated in modulating fungal growth under diverse environmental stress conditions, including oxidative stress [6], heat stress [7], and heavy-metal exposure [8]. However, the precise mechanisms by which NO governs fungal responses to Cd stress remain poorly understood.

Cd is a highly toxic environmental pollutant, posing significant risks to both ecosystem and human health [9,10,11]. Cd exposure has been shown to inhibit fungal growth [12], disrupt essential biological processes such as cell membrane integrity [13], enzyme activity [12,14], and DNA repair, while it also triggers cellular responses like oxidative stress and apoptosis [15,16]. Despite these observations, the molecular mechanisms through which fungi respond to Cd stress remain largely undefined.

The role of NO in heavy-metal toxicity is complex and context-dependent. On one hand, NO is known to mitigate growth inhibition under Cd stress by regulating redox balance [17] and enhancing stress tolerance [7]. On the other hand, NO can exacerbate metal-induced toxicity [3], suggesting that its effects vary according to environmental conditions and the specific nature of the stressor. In plants, NO has been demonstrated to alleviate the adverse effects of Cd and other heavy metals [18,19]. However, the interplay between NO signaling and Cd-induced stress in fungi has yet to be fully explored.

*Schizophyllum commune* is a widely distributed basidiomycete [20] that is not only edible but also has a variety of biological effects, including anti-inflammatory, antibacterial, antioxidant, and anticancer properties [21]. The signature molecule of *S. commune* is schizophyllan [22], which has been valued for its immunomodulatory, anti-tumor, and potential wound-healing properties [23]. The bioactive compounds produced by *S. commune* can also achieve antioxidant and anti-inflammatory effects by neutralizing free radicals and reducing oxidative stress [24]. In addition, *S. commune* plays a vital role in nutrient cycling by decomposing lignocellulosic materials through a variety of hydrolytic enzymes [25,26]. Therefore, once *S. commune* is contaminated with heavy metals, it will pose a significant risk to nutritional health and agricultural industrial applications.

In this study, we investigate the role of NO in mediating Cd-induced stress in the basidiomycete *S. commune* 20R-7-F01. Specifically, we focus on how NO signaling influences fungal growth under Cd stress. Our results indicate that Cd exposure triggers endogenous NO production, which activates cell wall hydrolases, leading to increased Cd accumulation in the fungal cell wall and inhibition of mycelial growth. These findings suggest that NO serves as a key mediator in the fungal response to Cd stress and may offer new insights for developing NO-based strategies to mitigate Cd pollution effects in fungi.

## 2. Materials and Methods

### 2.1. Fungal Strain

The strain *Schizophyllum commune* 20R-7-F01 (CGMCC 5.2202) used in this study was isolated from seafloor sediments at a depth of 1966 m during IODP Expedition 337 [27]. Detailed information about the habitat, minimal medium (MM), and culture conditions for this strain has been previously described [28,29].

### 2.2. Experimental Treatment

The growth of *S. commune* 20R-7-F01 was assessed by incubating 2 mm diameter mycelial plugs on solid minimal medium (MM) supplemented with 0–100 µM CdCl_2_ at 30 °C for 96 h. For liquid medium experiments, mycelia cultured in liquid MM for 72 h were washed 2–3 times with sterile deionized water. Subsequently, 1 g (wet weight) of mycelium was transferred to liquid MM containing 0–100 µM CdCl_2_ and different chemical treatments were added, including sodium nitroprusside (SNP, an NO donor, 300 µM), 2-(4-carboxyphenyl)-4,4,5,5-tetramethylimidazoline-1-oxyl-3-oxide (cPTIO, an NO scavenger, 200 µM), *N*^ω^-nitro-L-arginine methyl ester hydrochloride (L-NAME, a nitric oxide synthase (NOS) inhibitor, 100 µM), and sodium tungstate (Na_2_WO_4_, a nitrate reductase inhibitor, 300 µM), all of which were incubated at 30 °C for 96 h. All chemicals were purchased from Sigma-Aldrich (St. Louis, MO, USA). Mycelial growth and related indicators were measured after the incubation period.

### 2.3. NO Detection in Fungal Mycelium

Concentration of NO in *S. commune* mycelium was measured using the specific NO fluorescent probe DAF-2DA (4,5-diaminofluorescein diacetate, Sigma-Aldrich), as described by Wang et al. [30]. Briefly, mycelia were incubated with 2.5 μM DAF-2DA for 20 min at 37 °C, then washed 3–5 times with sterile deionized water, and placed on a microscope slide. NO concentration at the front end of the mycelium was observed using an Olympus BX53 fluorescence microscope (Evident, Tokyo, Japan) (excitation 450–490 nm, emission 500–530 nm). Fluorescence intensity was quantified using ImageJ (1.51n) software.

### 2.4. Quantification of NO Content

For quantitative analysis of NO content, the mycelia were washed 2–3 times with deionized water, then homogenized with 1 mL of a 50 mM acetic acid solution (pH 3.6) containing 4% (*w*/*v*) zinc acetate. The homogenate was centrifuged at 9500× *g* for 15 min at 4 °C. The NO content was determined according to the manufacturer’s protocol (A013-2-1, Nanjing Jiancheng Institute of Biological Engineering, Nanjing, China). Protein concentration was measured using the Bradford method [31]. Absorbance was recorded using an Infinite M200 Pro microplate reader (Tecan, Research Triangle Park, Durham, NC, USA).

### 2.5. Nitrate Reductase Activity Assay

Mycelia were ground in liquid nitrogen and homogenized in 1 mL of extraction buffer containing 50 mM HEPES-KOH (pH = 7.5), 5% (*v*/*v*) glycerol, 10 mM MgCl_2_, 1 mM dithiothreitol (DTT), 1 mM phenylmethylsulfonyl fluoride (PMSF), and 10 μM flavin adenine dinucleotide (FAD). The homogenate was centrifuged at 13,000× *g* for 20 min at 4 °C. The supernatant (200 μL) was divided into two tubes: one for protein determination, the other for nitrate reductase activity. For the assay, 500 μL of buffer (composed of 50 mM MOPS-KOH (pH 7.5), 10 mM KNO_3_, and 0.2 mM NADH) was added to the supernatant. The mixture was incubated at 25 °C for 30 min, then terminated by adding 50 μL of 0.5 M zinc acetate. Afterward, 500 μL of 1% (*w*/*v*) sulfanilamide and 500 μL of 0.02% (*w*/*v*) α-naphthylamine were added. The absorbance was measured at 540 nm after 15 min.

### 2.6. Cd Content Detection in Mycelium

To detect cadmium in mycelium, 50 μg of Leadmium™ Green AM dye (Thermo Fisher Scientific, Waltham, MA, USA) was dissolved in 50 μL of DMSO to prepare a stock solution. This was diluted 1:10 in saline (0.85% NaCl) to create a 10 μM working solution, which was protected from light. Ten mg of mycelium was incubated in 1 mL of the dye solution for 60 min in the dark. After washing 3–5 times with sterile deionized water to remove excess dye, the fluorescence was measured using an Olympus BX53 microscope with excitation at 450–490 nm and emission at 500–530 nm.

### 2.7. Cell Wall Extraction and Cd Analysis

One gram of mycelium was ground in liquid nitrogen, and 1.5 mL of 75% ethanol was added to create a homogenate. The sample was incubated on ice for 20 min, then centrifuged at 6520× *g* for 10 min. The precipitate was washed sequentially with 1 mL acetone, 1 mL mixture of methanol and chloroform (1/1, *v*/*v*), and 1 mL methanol, then freeze-dried and weighed. The dried cell wall was combusted at 550 °C for 20 h, dissolved in 2 mL HNO_3_ overnight, and diluted to 5 mL with deionized water. Cd content in the solution was measured using inductively coupled plasma atomic emission spectroscopy (ICP-AES, model PS-1000, Lowell, MA, USA) [32].

### 2.8. Chitinase Enzyme Activity Measurement

Chitinase enzyme activity was measured using a commercial detection kit (BC0825, Beijing Solabo Technology Co., Ltd., Beijing, China), following the manufacturer’s instructions [33].

### 2.9. Gene Expression Analysis by RT-qPCR

Gene expression levels were analyzed by real-time polymerase chain reaction (RT-qPCR) as described by [34]. Total RNA was extracted from 100 mg mycelium using RNAiso Plus (TaKaRa, Dalian, China) according to the manufacturer’s protocol. Reverse transcription was performed using the PrimeScript RT Reagent Kit with gDNA Eraser (TaKaRa) following the manufacturer’s instructions. SYBR Premix Ex Taq II (TaKaRa) was used for quantitative PCR analysis of chitinase-related genes, with *Actin* serving as the internal control. The primers used for gene expression analysis are listed in Supplementary Table S1.

### 2.10. Statistical Analysis

All experiments were repeated at least three times. Data are presented as mean and standard error. All data were analyzed using SPSS version 28.0.1.1 (Statistical Package for Social Sciences) and ImageJ software. For statistical analysis, we used Tukey’s test and least significant difference (LSD) test to determine the differences between treatments at *p* ≤ 0.05 level.

## 3. Results

### 3.1. Inhibition of S. commune Mycelial Growth by Cd

**Figure 1:**
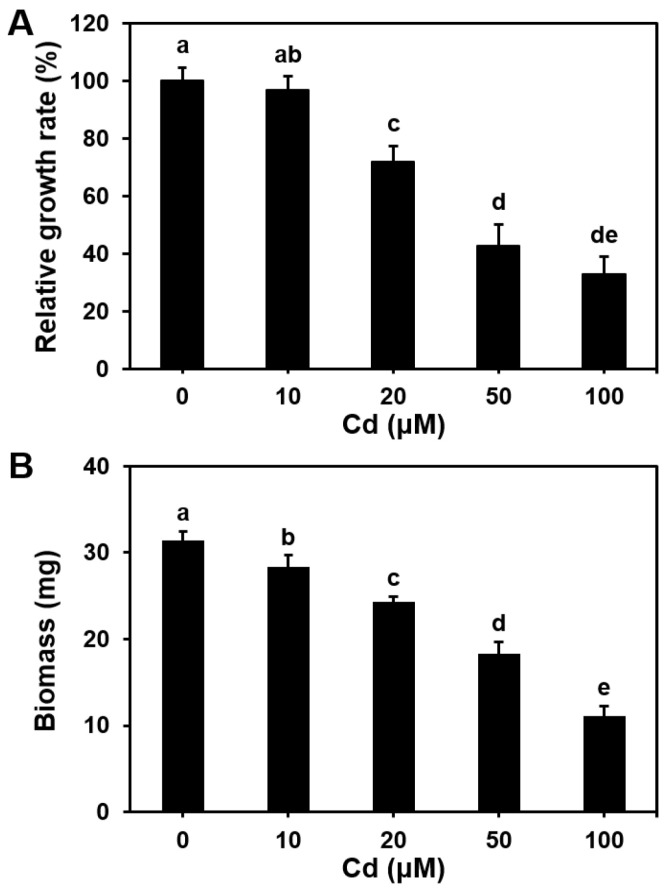
Cd inhibits the growth rate (**A**) and biomass (**B**) of *Schizophyllum commune*. Mycelial growth on solid (**A**) or liquid (**B**) MM containing 0–100 μM CdCl_2_. Relative growth rates are relative to the growth rates of mycelial in the presence of 0 μM Cd. All treatments were incubated at 30 °C for 96 h. Values are mean ± S.E (A, *n* = 20; B, *n* = 3). Different letters indicate statistically significant differences (*p* ≤ 0.05).

Mycelial growth of *S. commune* was assessed after culturing 2 mm diameter disks in solid MM supplemented with varying concentrations of CdCl_2_ for 96 h. Compared with the control (0 μM Cd), exposure to 10, 20, 50, and 100 μM Cd resulted in a 3%, 28%, 57%, and 67% inhibition of mycelial growth, respectively (Figure 1A). Similarly, when mycelia were cultured in liquid MM under identical conditions, a concentration-dependent decrease in biomass was observed (Figure 1B). These results indicated that *S. commune* growth is inhibited by Cd and the inhibitory effect increased with the increasing Cd concentration.

### 3.2. Effect of Cd Stress on Endogenous NO Production in S. commune

**Figure 2:**
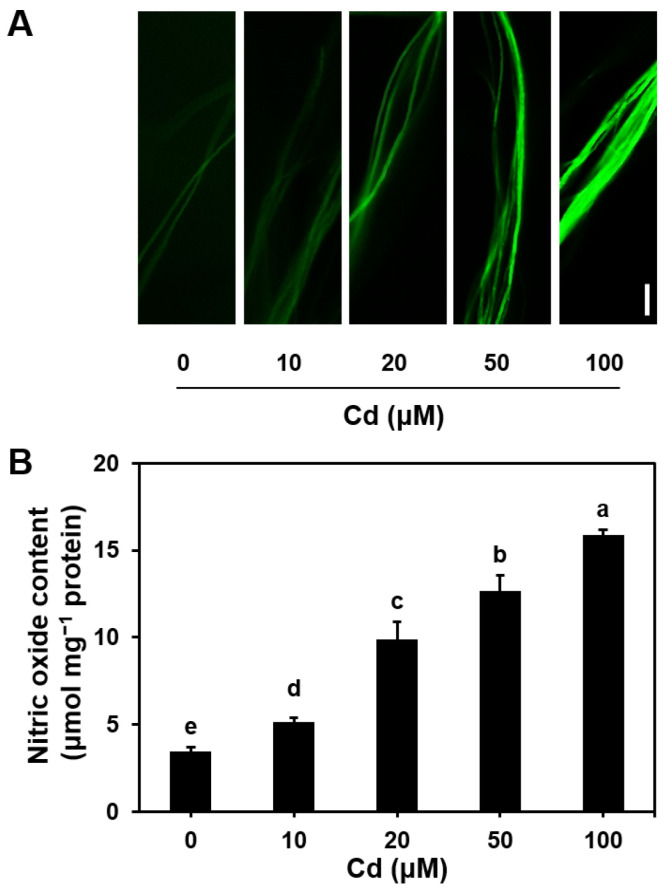
NO production in *S. commune* mycelium under exposure to various concentrations of CdCl_2_. (**A**) Fluorescence intensity diagram of NO production detected by NO-specific fluorescent probe DAF-2DA in mycelium treated with different Cd concentrations. (**B**) Quantitative analysis of NO content in mycelium. All mycelium were exposed to 0, 10, 20, 50, or 100 μM CdCl_2_ in solid liquid MM medium at 30 °C for 96 h. μmol mg^−1^ protein indicates the NO content per mg of protein. Values are mean ± S.E (*n* = 3). Different letters indicate significant differences (*p* ≤ 0.05). Bar = 10 μm.

The effect of Cd stress on the production of endogenous NO in *S. commune* mycelium was assessed using the NO-specific fluorescent probe DAF-2DA (Figure 2A). After 96 h of exposure to 10, 20, 50, or 100 μM Cd, a concentration-dependent increase in the fluorescence intensity of endogenous NO was observed, compared to the control (0 μM Cd). Specifically, NO levels under exposure to 10, 20, 50, and 100 μM Cd were 1.50, 2.87, 3.69, and 4.62 times higher than those of the control, respectively (Figure 2B). Based on these results, 100 μM Cd was selected for subsequent experiments. These findings demonstrated that Cd exposure significantly enhanced NO production in *S. commune* mycelium.

### 3.3. Endogenous NO Synthesis Pathway in S. commune Under Cd Stress

**Figure 3:**
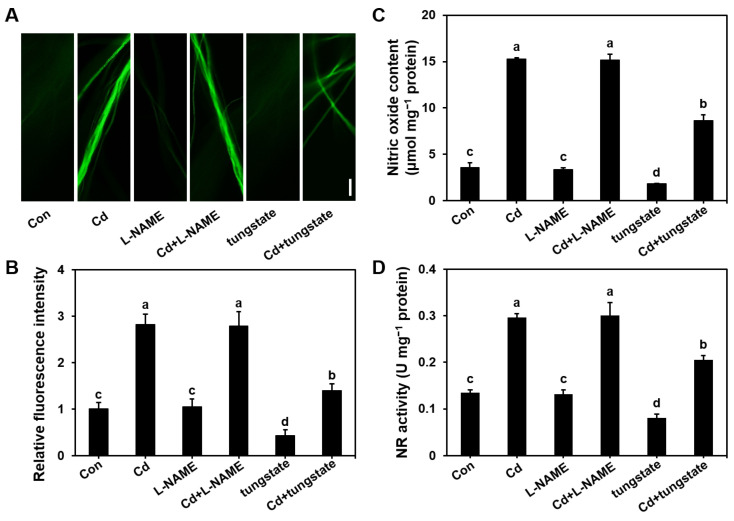
NO content and nitrate reductase (NR) activity in *S. commune* mycelium cultured with Cd in the presence and absence of NOS and NR inhibitors. (**A**) Fluorescence intensity image of NO in mycelium under different treatments detected by the NO-specific fluorescent probe DAF-2DA, and (**B**) digital fluorescence intensity. (**C**) NO content. (**D**) NR activity. Mycelium was cultured in liquid MM medium with 0 or 100 μM Cd, in the presence or absence of 100 μM L-NAME (NOS inhibitor) or 300 μM tungstate (NR inhibitor), at 30 °C for 96 h. Con with the control (0 μM Cd). μmol mg^−1^ protein indicates the NO content per mg of protein. U represents the amount of enzyme required to convert 1 μmol of substrate in 1 min. U μmol mg^−1^ protein represents the enzyme activity per mg of protein. Values are mean ± S.E (*n* = 3). Different letters indicate significant differences (*p* ≤ 0.05). Bar = 10 μm.

To investigate the endogenous source of NO production in *S. commune* under Cd stress, mycelium was cultured in MM medium supplemented with the NOS inhibitor L-NAME and the nitrate reductase (NR) inhibitor tungstate at 30 °C for 96 h. Compared to the control (0 μM Cd), Cd exposure significantly increased both the NO content (Figure 3A–C) and NR activity (Figure 3D), by 4.24- and 2.20-folds, respectively. However, treatment with L-NAME, either alone or in combination with Cd, did not significantly alter the NO levels or NR activity (Figure 3). These findings indicated that the increase in endogenous NO under Cd stress was not mediated by NOS, which was consistent with the absence of NOS-related genes in the *S. commune* genome [35].

In contrast, the addition of tungstate, an NR inhibitor, resulted in a significant reduction in both the NO content and the NR activity compared to Cd treatment alone. Specifically, the NO content was reduced by 50%, and the NR activity decreased by 31% (Figure 3). Additionally, a strong positive linear correlation between the NR activity and the NO content was observed under Cd stress (Figure S1). These results strongly suggested that the Cd-induced synthesis of NO in *S. commune* was primarily mediated through NR, rather than NOS-dependent pathways.

### 3.4. Effect of NO Modulation on Cd Accumulation in S. commune Mycelium

**Figure 4:**
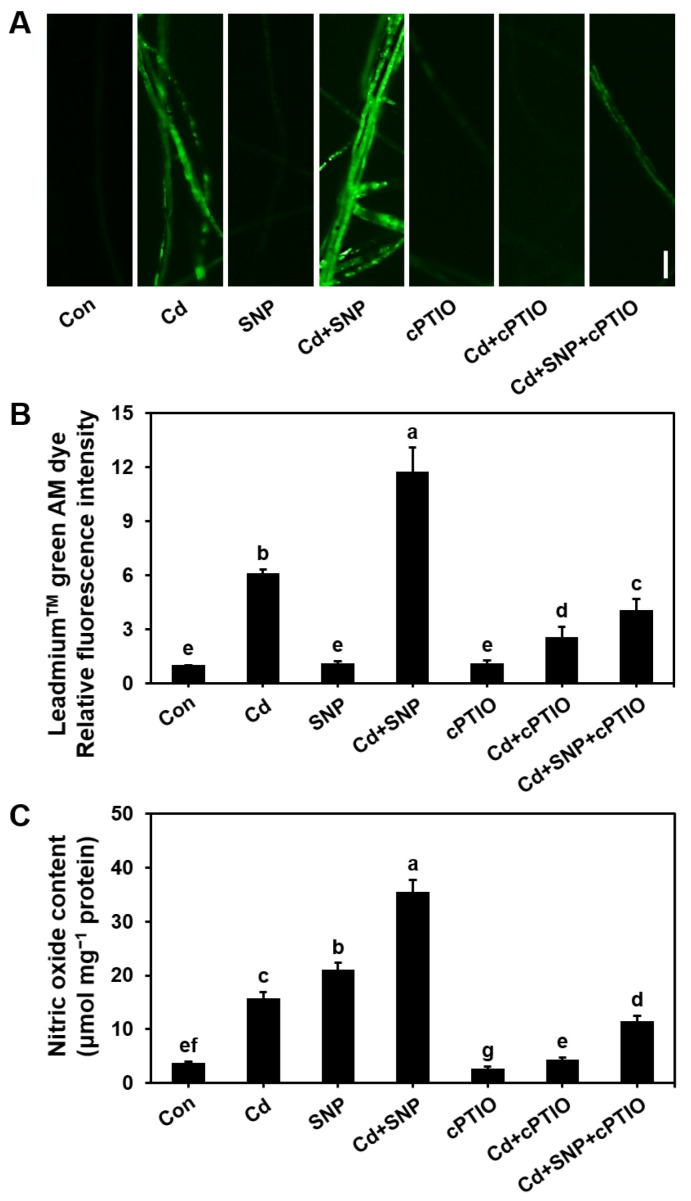
Effect of NO modulation on Cd accumulation in mycelium. (**A**) Image of Cd distribution in mycelium, measured using the Cd-specific fluorescent probe Leadmium ^TM^ green AM dye; (**B**) relative fluorescence intensity in the images; (**C**) NO content. Mycelium was cultured in liquid MM medium with 0 or 100 μM Cd, in the presence or absence of 300 μM SNP (NO donor) or 200 μM cPTIO (NO scavenger), at 30 °C for 96 h. Con with the control (0 μM Cd). μmol mg^−1^ protein indicates the NO content per mg of protein. Values are mean ± S.E (*n* = 6). Different letters indicate significant differences (*p* ≤ 0.05). Bar = 10 μm.

To investigate the role of endogenous NO in Cd accumulation in mycelium, we cultured *S. commune* mycelium at 30 °C for 96 h in liquid MM medium, with or without CdCl_2_, SNP (NO donor), and cPTIO (NO scavenger). The results demonstrated that SNP significantly increased both the NO levels and Cd accumulation in the mycelium compared to Cd stress alone. In contrast, cPTIO alone or together with cPTIO and SNP markedly reduced NO production and inhibited Cd accumulation (Figure 4). These findings suggested that overproduction of NO promotes Cd accumulation in mycelium.

### 3.5. Cd-Induced NO Accumulation Increased Chitinase Activity in S. commune

**Figure 5:**
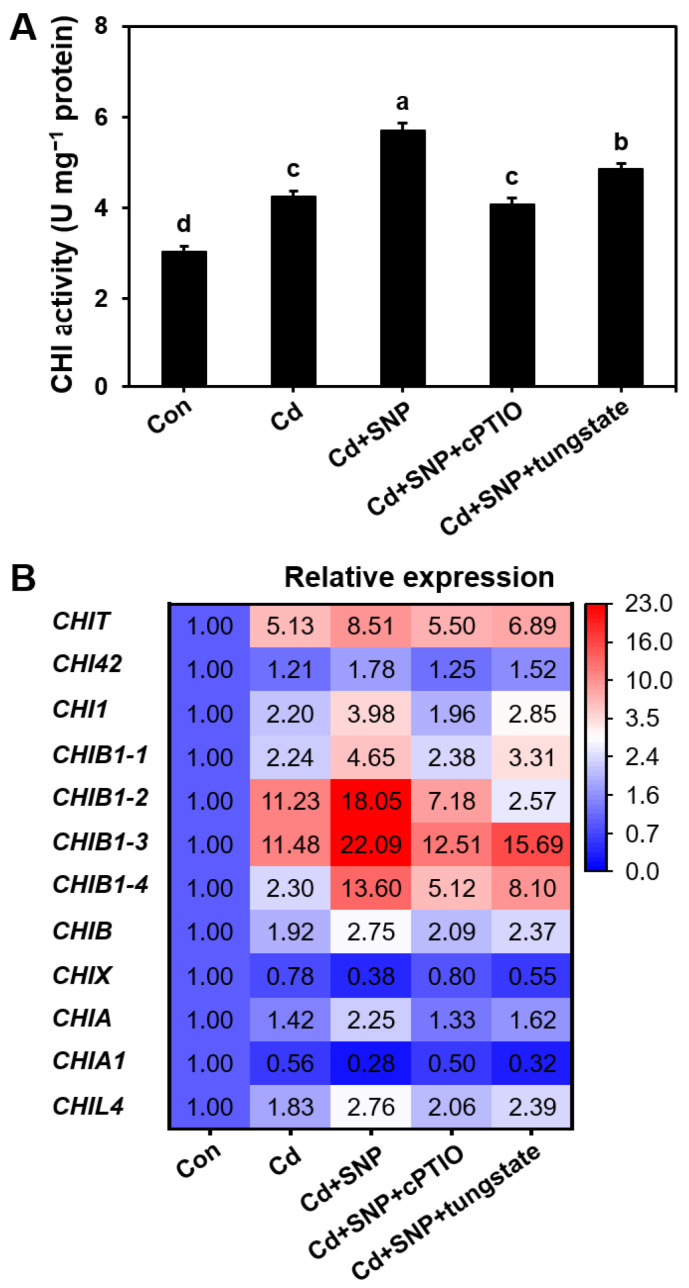
Effect of Cd and Cd-induced NO on fungal chitinase activity (**A**) and the relative expression of chitinase-encoding genes (**B**). Mycelium was cultured in liquid MM medium with 0 or 100 μM Cd, in the presence or absence of 300 μM SNP (NO donor), 200 μM cPTIO (NO scavenger), or 300 μM tungstate (NR inhibitor), at 30 °C for 96 h. Con with the control (0 μM Cd). U represents the amount of enzyme required to convert 1 μmol of substrate in 1 min. U μmol mg^−1^ protein represents the enzyme activity per mg of protein. Values are mean ± S.E (*n* = 3). Different letters indicate significant differences (*p* ≤ 0.05).

Exposure to 100 μM Cd significantly increased chitinase activity in the mycelium of *S. commune*, with a 1.40-folds enhancement compared to the control (Figure 5A). The addition of the NO donor SNP further increased chitinase activity by 1.88 times. However, when the specific NO scavenger cPTIO was present, chitinase activity was reduced by 28.4%, indicating that NO played a critical role in mediating the Cd-induced enhancement of chitinase activity. Similarly, in the presence of the NO synthesis inhibitor tungstate, chitinase activity was significantly decreased by 14.8%, further verifying the involvement of NO in the regulation of chitinase activity (Figure 5A). In addition, there was a strong positive linear correlation between CHI activity and NO content (Figure S2).

RT-qPCR analysis revealed that Cd exposure markedly upregulated the expression of several chitinase-related genes, including *CHIT*, *CHI1*, *CHIB1-2*, *CHIB1-3*, *CHIB1-4*, *CHIB*, *CHIX*, *CHIA*, *CHIA1*, and *CHIL4*, while *CHI42* and *CHIB1-1* were significantly downregulated (Figure 5B). These transcriptional changes suggested that Cd-induced NO accumulation enhanced chitinase activity allowed fungal cell wall hydrolysis.

### 3.6. Cd Accumulation in the Fungal Cell Wall and Its Impact on Hyphal Growth

**Figure 6:**
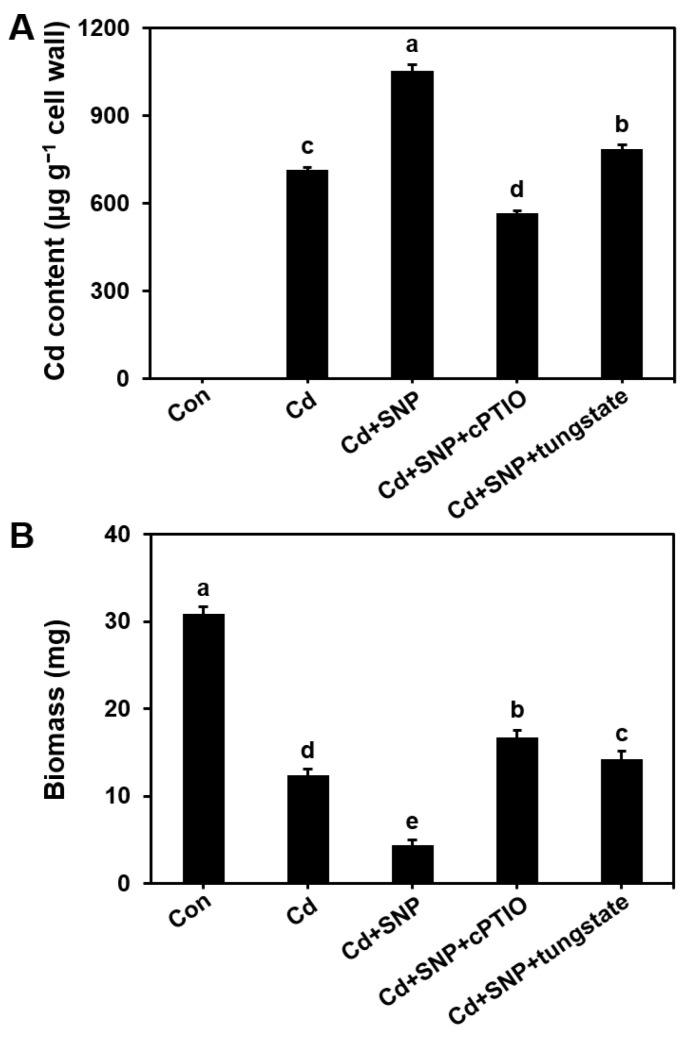
Accumulation of Cd in the cell wall (**A**) and mycelial biomass (**B**) of *S. commune* under exposure to Cd in the presence of NO modulators. Mycelium was cultured in liquid MM medium with 0 or 100 μM Cd, in the presence or absence of 300 μM SNP (NO donor), 200 μM cPTIO (NO scavenger), or 300 μM tungstate (NR inhibitor), at 30 °C for 96 h. Con with the control (0 μM Cd). μg g^−1^ cell wall represents the Cd content per g of cell wall. Values are mean ± S.E (*n* = 3). Different letters indicate significant differences (*p* ≤ 0.05).

To investigate the accumulation of Cd in the fungal cell wall and its potential impact on mycelial growth, we exposed *S. commune* to Cd in MM medium, with or without the addition of SNP, cPTIO, and sodium tungstate. The control group showed no detectable Cd accumulation in the cell wall. However, Cd treatment alone resulted in significant accumulation, with a concentration of 716 µg g^−1^ in the mycelial cell wall. The presence of SNP increased Cd accumulation to 1052 µg g^−1^. In contrast, the presence of cPTIO reduced Cd accumulation by 45.6% and 21.7% compared to the treatment with and without the addition of SNP. The combined exposure to Cd, SNP, and tungstate resulted in a higher Cd accumulation than the exposure to Cd alone, which was still 24.1% lower than the exposure to Cd and SNP (Figure 6A).

Interestingly, mycelium growth was negatively correlated with the Cd accumulation in the cell wall. Excessive NO accumulation induced by SNP enhanced Cd retention in the cell wall, which subsequently inhibited mycelial growth. These results suggested that NO-mediated Cd accumulation in the cell wall negatively impacted the mycelial growth (Figure 6B).

## 4. Discussion

Cd is a highly toxic heavy metal that significantly impairs the growth and metabolism of fungi, consequently posing serious risks to environmental health and ecological stability [36]. In this study, we explored the response of *S. commune* mycelium to Cd stress, particularly focused on the role of NO as a signaling molecule. Our findings highlight the critical role of NO in regulating fungal responses to Cd toxicity, particularly through its influence on cell wall hydrolysis and Cd accumulation.

The key observation in this study was that Cd exposure increased NO production (Figure 2) in *S. commune* mycelium, primarily through the activation of the nitrate reductase (NR) pathway. Inhibition of NR activity by tungstate or scavenger of NO by the NO scavenger cPTIO can effectively reduce NO levels and mitigate the toxicity of Cd (Figure 3 and Figure 5).

These results suggested that NR-mediated NO production played a pivotal role in the fungal response to Cd stress. This finding was in agreement with previous reports that demonstrated NO as a pivotal mediator of stress responses in fungi, particularly in the context of heavy-metal exposure [37,38].

NO plays a crucial role in regulating fungal cell wall integrity, a key mechanism underlying NO toxic effects in response to Cd stress. The fungal cell wall, which is primarily composed of polysaccharides such as chitin and β-glucans [39], serves as a primary defense against external stresses, including heavy metals. Our findings demonstrated that NO significantly enhanced chitinase activity in *S. commune* (Figure 5). Chitinases are critical enzymes involved in the turnover and remodeling of the fungal cell wall, degrading chitin into smaller oligosaccharides [40]. In this study, we showed that NO-induced chitinase activity contributed to the increased Cd accumulation within the cell wall of *S. commune* (Figure 5), thereby inhibiting fungal growth.

Cd accumulation in fungal cell walls can effectively reduce the bioavailability of Cd and reduce environmental pollution [41]. Fungi with strong Cd accumulation in cell walls can occupy ecological niches in Cd-contaminated soils and affect the structure of microbial communities [42]. Cd accumulation in mycorrhizal fungal cell walls can reduce plant absorption of Cd and alleviate heavy-metal toxicity. At the same time, Cd accumulation in fungal cell walls helps prevent its spread and reduce the risk of entering the food chain [43]. Overall, Cd accumulation in fungal cell walls plays an important role in buffering and repairing Cd-contaminated environments. At the same time, the accumulation of Cd in the cell wall is also crucial for Cd toxicity and can inhibit fungal growth. Our study also revealed that inhibiting NO production, either through NR activity inhibition or the application of cPTIO, reduced Cd accumulation in both the mycelium (Figure 4) and the cell wall (Figure 6). These findings highlighted the importance of NO in regulating Cd accumulation in the fungal cell wall and underscored the role of NO in Cd cytotoxicity.

**Figure 7:**
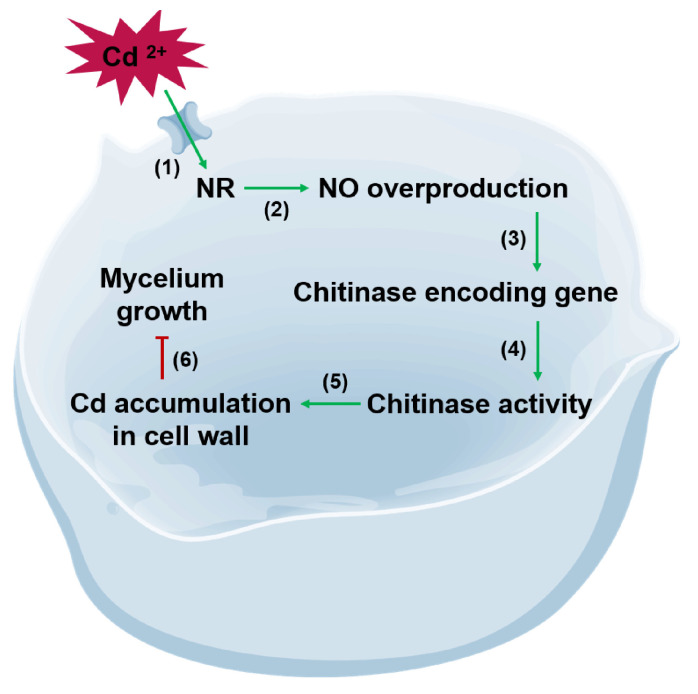
Proposed mechanism of Cd toxicity in inhibiting *S. commune* 20R-7-F01 growth. Cd exposure activates NR (1), leading to the production of NO (2). Elevated endogenous NO levels subsequently enhance the expression of chitinase genes (3) and increase chitinase activity (4), which facilitates the accumulation of Cd in the fungal cell wall (5). This process ultimately contributes to the inhibition of mycelial growth (6). Green arrows denote positive regulatory effects, while red-capped line indicates negative regulatory effects.

Collectively, these findings suggested that Cd-induced NO production played a crucial role in the response of *S. commune* to heavy-metal stress by enhancing chitinase activity and Cd accumulation in the fungal cell wall, which ultimately inhibited the fungal growth (Figure 7). Therefore, modulating NO synthesis and chitinase activity presented a potential strategy to mitigate the adverse effects of Cd in fungi. Subsequent studies can edit NR and CHI-related coding genes by CRISPR-Cas9 to reduce Cd accumulation in fungal cell walls. Future studies will focus on manipulating NR-mediated NO production, which may provide an effective approach to enhance fungal tolerance to heavy-metal pollution and mitigate heavy-metal accumulation in edible fungi.

## Data Availability

The original contributions presented in this study are included in the article/Supplementary Materials. Further inquiries can be directed to the corresponding author.

## Supplementary Materials

The following supporting information can be downloaded at: https://www.mdpi.com/article/10.3390/microorganisms13030470/s1, Figure S1: correlation between the content of NO and the activity of NR in the mycelium of *S. commune*; Figure S2: correlation between NO content and CHI activity in *S. commune* mycelia. Table S1: primers used in this study.

## References

[1] Gantner B.N., LaFond K.M., Bonini M.G. (2020). Nitric oxide in cellular adaptation and disease. Redox Biol..

[2] Sharma A., Soares C., Sousa B., Martins M., Kumar V., Shahzad B., Sidhu G.P., Bali A.S., Asgher M., Bhardwaj R. (2020). Nitric oxide-mediated regulation of oxidative stress in plants under metal stress: A review on molecular and biochemical aspects. Physiol. Plant..

[3] Zhang J., Li D., Wei J., Ma W., Kong X., Rengel Z., Chen Q. (2019). Melatonin alleviates aluminum-induced root growth inhibition by interfering with nitric oxide production in *Arabidopsis*. Environ. Exp. Bot..

[4] Yu N.-N., Park G. (2024). Nitric Oxide in Fungi: Production and Function. J. Fungi.

[5] Zhao Y., Lim J., Xu J., Yu J.H., Zheng W. (2020). Nitric oxide as a developmental and metabolic signal in filamentous fungi. Mol. Microbiol..

[6] Yu Y., Yang Z., Guo K., Li Z., Zhou H., Wei Y., Li J., Zhang X., Harvey P., Yang H. (2015). Oxidative damage induced by heat stress could be relieved by nitric oxide in *Trichoderma harzianum* LTR-2. Curr. Microbiol..

[7] Kong W., Huang C., Chen Q., Zou Y., Zhang J. (2012). Nitric oxide alleviates heat stress-induced oxidative damage in *Pleurotus eryngii var*. tuoliensis. Fungal Genet. Biol..

[8] Chiang K.T., Shinyashiki M., Switzer C.H., Valentine J.S., Gralla E.B., Thiele D.J., Fukuto J.M. (2000). Effects of nitric oxide on the copper-responsive transcription factor Ace1 in *Saccharomyces cerevisiae*: Cytotoxic and cytoprotective actions of nitric oxide. Arch. Biochem. Biophys..

[9] Suhani I., Sahab S., Srivastava V., Singh R.P. (2021). Impact of cadmium pollution on food safety and human health. Curr. Opin. Toxicol..

[10] Khan Z., Elahi A., Bukhari D.A., Rehman A. (2022). Cadmium sources, toxicity, resistance and removal by microorganisms-A potential strategy for cadmium eradication. J. Saudi Chem. Soc..

[11] Waalkes M.P., Misra R.R., Chang L.W., Suzuli T. (2023). Cadmium carcinogenicity and genotoxicity. Toxicology of Metals.

[12] El-Mahdy O.M., Mohamed H.I., Mogazy A.M. (2021). Biosorption effect of *Aspergillus niger* and *Penicillium chrysosporium* for Cd-and Pb-contaminated soil and their physiological effects on *Vicia faba* L.. Environ. Sci. Pollut. Res..

[13] Volesky B., May H., Holan Z. (1993). Cadmium biosorption by *Saccharomyces cerevisiae*. Biotechnol. Bioeng..

[14] Baldrian P., Gabriel J. (2003). Lignocellulose degradation by *Pleurotus ostreatus* in the presence of cadmium. FEMS Microbiol. Lett..

[15] Ma Y., Su Q., Yue C., Zou H., Zhu J., Zhao H., Song R., Liu Z. (2022). The effect of oxidative stress-induced autophagy by cadmium exposure in kidney, liver, and bone damage, and neurotoxicity. Int. J. Mol. Sci..

[16] Zheng J., Zhuo L., Ran D., Ma Y., Luo T., Zhao H., Song R., Zou H., Zhu J., Gu J. (2020). Cadmium induces apoptosis via generating reactive oxygen species to activate mitochondrial p53 pathway in primary rat osteoblasts. Toxicology.

[17] Abdalmegeed D., Zhao G., Cheng P., Bhat J.A., Khattak W.A., Ali M.G., Alnadari F., Ali I., Ali Q., Korma S.A. (2021). The Importance of Nitric Oxide as the Molecular Basis of the Hydrogen Gas Fumigation-Induced Alleviation of Cd Stress on Ganoderma lucidum. J. Fungi.

[18] Gallego S.M., Kogan M.J., Azpilicueta C.E., Peña C., Tomaro M.L. (2005). Glutathione-mediated antioxidative mechanisms in sunflower (*Helianthus annuus* L.) cells in response to cadmium stress. Plant Growth Regul..

[19] Yang L.-T., Qi Y.-P., Chen L.-S., Sang W., Lin X.-J., Wu Y.-L., Yang C.-J. (2012). Nitric oxide protects sour pummelo (*Citrus grandis*) seedlings against aluminum-induced inhibition of growth and photosynthesis. Environ. Exp. Bot..

[20] Mišković J., Rašeta M., Krsmanović N., Karaman M. (2023). Update on mycochemical profile and selected biological activities of genus *Schizophyllum* Fr. 1815. Microbiol. Res..

[21] Abd Razak D.L., Abd Ghani A., Lazim M.I.M., Khulidin K.A., Shahidi F., Ismail A. (2024). *Schizophyllum commune* (Fries) mushroom: A review on its nutritional components, antioxidative, and anti-inflammatory properties. Curr. Opin. Food Sci..

[22] Zhang Y., Kong H., Fang Y., Nishinari K., Phillips G.O. (2013). Schizophyllan: A review on its structure, properties, bioactivities and recent developments. Bioact. Carbohydr. Diet. Fibre.

[23] Desiderio A., Goppa L., Santambrogio C., Brocca S., Buratti S., Girometta C.E., Sarkar M., Venuti M.T., Savino E., Rossi P. (2025). Improving the Proteome-Mining of *Schizophyllum commune* to Enhance Medicinal Mushroom Applications. J. Fungi.

[24] Mišković J., Karaman M., Rašeta M., Krsmanović N., Berežni S., Jakovljević D., Piattoni F., Zambonelli A., Gargano M.L., Venturella G. (2021). Comparison of two *Schizophyllum commune* strains in production of acetylcholinesterase inhibitors and antioxidants from submerged cultivation. J. Fungi.

[25] Ahmad N., Aslam S., Hussain N., Bilal M., Iqbal H.M. (2023). Transforming lignin biomass to value: Interplay between ligninolytic enzymes and lignocellulose depolymerization. BioEnergy Res..

[26] Kumar A., Bharti A.K., Bezie Y. (2022). *Schizophyllum commune*: A fungal cell-factory for production of valuable metabolites and enzymes. BioResources.

[27] Liu C.H., Huang X., Xie T.N., Duan N., Xue Y.R., Zhao T.X., Lever M.A., Hinrichs K.U., Inagaki F. (2017). Exploration of cultivable fungal communities in deep coal-bearing sediments from∼ 1.3 to 2.5 km below the ocean floor. Environ. Microbiol..

[28] Zain Ul Arifeen M., Ma Z.J., Wu S., Liu J.Z., Xue Y.R., Liu C.H. (2021). Effect of oxygen concentrations and branched-chain amino acids on the growth and development of sub-seafloor fungus, *Schizophyllum commune* 20R-7-F01. Environ. Microbiol..

[29] Zhao M., Li D., Liu J., Fang J., Liu C. (2024). Pressure-tolerant survival mechanism of *Schizophyllum commune* 20R-7-F01 isolated from deep sediments 2 kilometers below the seafloor. Front. Mar. Sci..

[30] Wang Y., Zhang X., Lu C., Li X., Zhou J., Wang J. (2022). Lanthanum: A novel inducer for enhancement of fungal laccase production by *Shiraia bambusicola*. J. Rare Earths.

[31] Bradford M.M. (1976). A rapid and sensitive method for the quantitation of microgram quantities of protein utilizing the principle of protein-dye binding. Anal. Biochem..

[32] Li D., Xiao S., Ma W.-N., Peng Z., Khan D., Yang Q., Wang X., Kong X., Zhang B., Yang E. (2020). Magnesium reduces cadmium accumulation by decreasing the nitrate reductase-mediated nitric oxide production in *Panax notoginseng* roots. J. Plant Physiol..

[33] Jiang N., Wang L., Jiang D., Wang M., Liu H., Yu H., Yao W. (2022). Transcriptomic analysis of inhibition by eugenol of ochratoxin A biosynthesis and growth of *Aspergillus carbonarius*. Food Control.

[34] Pfaffl M.W. (2001). A new mathematical model for relative quantification in real-time RT–PCR. Nucleic Acids Res..

[35] Liu X., Huang X., Chu C., Xu H., Wang L., Xue Y., Muhammad Z.U.A., Inagaki F., Liu C. (2022). Genome, genetic evolution, and environmental adaptation mechanisms of *Schizophyllum commune* in deep subseafloor coal-bearing sediments. Iscience.

[36] Liu J., Fu P., Wang L., Lin X., Enayatizamir N. (2022). A fungus (*Trametes pubescens*) resists cadmium toxicity by rewiring nitrogen metabolism and enhancing energy metabolism. Front. Microbiol..

[37] Li Q., Huang W., Xiong C., Zhao J. (2018). Transcriptome analysis reveals the role of nitric oxide in *Pleurotus eryngii* responses to Cd^2+^ stress. Chemosphere.

[38] Guo S., Yao Y., Zuo L., Shi W., Gao N., Xu H. (2016). Enhancement of tolerance of *Ganoderma lucidum* to cadmium by nitric oxide. J. Basic Microbiol..

[39] Seidl-Seiboth V., Ihrmark K., Druzhinina I., Karlsson M., Gupta S.M., Herrera-Estrella A., Upadhyay R.S., Druzhinina I., Tuohy M.G. (2014). Molecular evolution of Trichoderma chitinases. Biotechnology and biology of Trichoderma.

[40] Hartl L., Zach S., Seidl-Seiboth V. (2012). Fungal chitinases: Diversity, mechanistic properties and biotechnological potential. Appl. Microbiol. Biotechnol..

[41] Mubeen S., Ni W., He C., Yang Z. (2023). Agricultural strategies to reduce cadmium accumulation in crops for food safety. Agriculture.

[42] Oggerin M., Del Moral C., Rodriguez N., Fernandez-Gonzalez N., Martínez J.M., Lorca I., Amils R. (2024). Metal tolerance of Río Tinto fungi. Front. Fungal Biol..

[43] Crișan I., Balestrini R., Pagliarani C. (2024). The current view on heavy metal remediation: The relevance of the plant interaction with arbuscular mycorrhizal fungi. Plant Stress.

