# Supplementary material for: Nitric Oxide-Mediated Regulation of Chitinase Activity and Cadmium Sequestration in the Response of Schizophyllum commune to Cadmium Stress
Source: Microorganisms. 2025 Feb 20;13(3):470. doi: 10.3390/microorganisms13030470 (PMC11944285; doi:10.3390/microorganisms13030470)
Supplement: Supplementary file 1 [file microorganisms-13-00470-s001.zip › microorganisms-3469026-supplementary.pdf]

**Supplementary Information for:**

# Nitric Oxide-Mediated Regulation of Chitinase Activity and Cadmium Sequestration in the Response of *Schizophyllum commune* to Cadmium Stress

**Dongxu Li [1], Chen Chu [1], Mengshi Zhao [1], Suying Hou [2], Rong Ji [3] and Changhong Liu [1,*]**

[1] State Key Laboratory of Pharmaceutical Biotechnology, School of Life Sciences, Nanjing University, Nanjing 210023, China; laolang_2012@163.com (D.L.); mg1930112@smail.nju.edu.cn (C.C.); 602022300055@smail.nju.edu.cn (M.Z.)

[2] College of Life Sciences, Yunnan University, Kunming 650500, China; housuying1008@163.com

[3] State Key Laboratory of Pollution Control and Resource Reuse, School of Environment, Nanjing University, Nanjing 210023, China; ji@nju.edu.cn

* Correspondence: chliu@nju.edu.cn

**Figure S1**

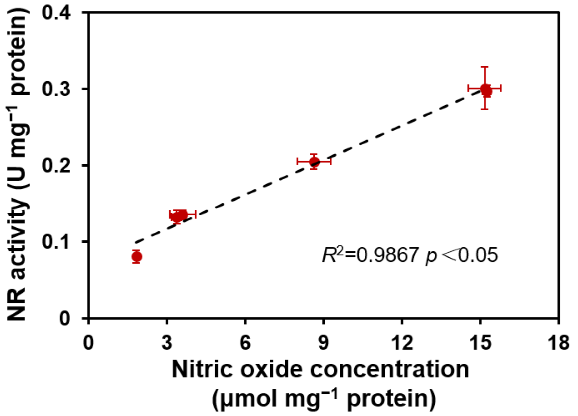


**Figure S1**. Correlation between the content of NO and the activity of NR in the mycelium of *S. commune*. Mycelium was cultured in liquid MM medium with 0 or 100 μM Cd, in the presence or absence of 100 μM L-NAME (NOS inhibitor) or 300 μM tungstate (NR inhibitor), at 30 °C for 96 h. Con with the control (0 μM Cd). Detection of NO content and NR activity in mycelium. U represents the amount of enzyme required to convert 1 μmol of substrate in 1 minute. U μmol $mg^{-1}$ protein represents the enzyme activity per mg of protein. Values are mean ± S.E ($n$ = 3).

**Figure S2**

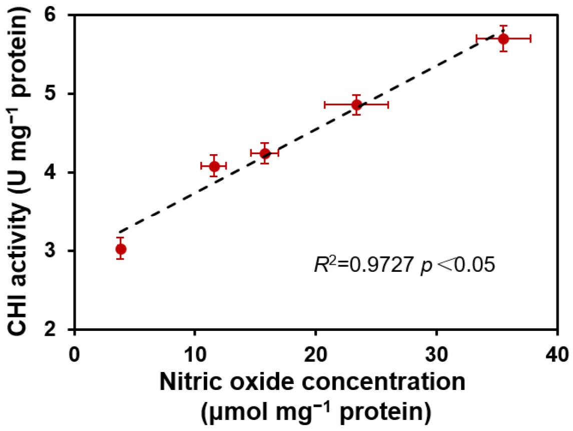


**Figure S2**. Correlation between NO content and CHI activity in *S. commune* mycelia. Mycelia were cultured in liquid MM medium containing 0 or 100 μM Cd, in the presence or absence of 300 μM SNP (NO donor), 200 μM cPTIO (NO scavenger), or 300 μM tungstate (NR inhibitor) at 30 °C for 96 h. Con with the control (0 μM Cd). Detection of NO content and CHI activity in mycelium. U represents the amount of enzyme required to convert 1 μmol of substrate in 1 minute. U μmol $mg^{-1}$ protein represents the enzyme activity per mg of protein. μmol $mg^{-1}$ protein indicates the NO content per mg of protein. Values are mean ± S.E ($n$ = 3).

**Table S1.** Primers used in this study

| Primer | Sequence (5′-3′) | Application |
| --- | --- | --- |
| Actin-F | TGGTATCCTCACGTTGAAGTA | Real-time RT-PCR (internal control) |
| *Actin*-R | GTGTGGTGCCAGATCTT | Real-time RT-PCR (internal control) |
| *CHIT*-F | CCGTCTTCGTGAATGAGAT | Real-time RT-PCR |
| *CHIT*-R | CGCACTGATTGTTGTAGAAT | Real-time RT-PCR |
| *CHI42*-F | GGCTATGGATTCTGCTCTG | Real-time RT-PCR |
| *CHI42*-R | CGGTGCTAATATCGCTGAT | Real-time RT-PCR |
| *CHI1*-F | TCTACAACAACTACTGCTCTG | Real-time RT-PCR |
| *CHI1*-R | CGATGACCTGCTGAACTG | Real-time RT-PCR |
| *CHIB1-1*-F | GCTACCTTGACATCTGGAA | Real-time RT-PCR |
| *CHIB1-1*-R | GGACACCTACGACAATCTT | Real-time RT-PCR |
| *CHIB1-2*-F | CGTGGCTTCCTACTCCTA | Real-time RT-PCR |
| *CHIB1-2*-R | ATGGTTCTGCGTGCTATC | Real-time RT-PCR |
| *CHIB1-3*-F | ACATCGTGAAGCAGAAGG | Real-time RT-PCR |
| *CHIB1-3*-R | GCAGGAGGAGCAGTAGTA | Real-time RT-PCR |
| *CHIB1-4*-F | GACCTCCAGAAGCAGAAG | Real-time RT-PCR |
| *CHIB1-4*-R | GAGTAGTCGTAAGCCATCA | Real-time RT-PCR |
| *CHIB*-F | AGGAGAACGGTATCAAGGT | Real-time RT-PCR |
| *CHIB*-R | CAGCGTGATGAGGAAGTC | Real-time RT-PCR |
| *CHIX*-F | CGGCGACTACATCATCAC | Real-time RT-PCR |
| *CHIX*-R | AGGAGGAGGTGTTGAGGA | Real-time RT-PCR |
| *CHIA*-F | TTGATGACACCAGCAAGG | Real-time RT-PCR |
| *CHIA*-R | GAGGAAGGAGAGGAAGTTG | Real-time RT-PCR |
| *CHIA1*-F | TCCTCCATCTAATGTCTCCT | Real-time RT-PCR |
| *CHIA1*-R | GTGACGGTCTTGACGAAT | Real-time RT-PCR |
| *CHIL4*-F | ACCAATGTAACCGCCTTC | Real-time RT-PCR |
| *CHIL4*-R | GTTGAACGCAGAGGTCTT | Real-time RT-PCR |
